# Action for Malpractice

**Published:** 1899-01

**Authors:** 


### Action for Malpractice.

Dr. G. W. Thompson (Cleveland *Medical Gazette,* November), in a thoughtful and interesting paper on this subject, says that the most dangerous question that confronts us as a profession to-day is that of liability to the public from a medico-legal point of view. The great misfortune is that we are powerless to defend ourselves so far as the law is concerned. There is no law in any of the statute books that justifies us in entering into a written contract with persons we are called upon to treat. In all other classes of business, contracts are written and signed by the parties who may enter into them, and each party to the contract is held responsible for the faithful carrying out of its covenants.

The physician and surgeon have no protection whatever in this respect that can be relied upon in the statutes. There can be no contract entered into between a physician and his patient that is binding in law. This being true, when we are called upon to treat and take charge of a case, it matters not of what character or what the prospects are for an unfavorable termination, we must do so at our own risk.

As regards the actually implied contract between the physician and his patient, Dr. Thompson points out that the law implies that a physician or surgeon, without a special contract for that purpose, is never considered as warranting a cure; that his contract as implied in law is, that he possesses that reasonable degree of learning, skill, and experience which is ordinarily possessed by others of his profession; that he will use reasonable and ordinary care and diligence in the treatment of cases committed to his care; that he will use his best judgment in all cases of doubt as to the best course of treatment; that he is not responsible for want of success unless it is proved to result from want of ordinary skill, or from want of ordinary care and attention; that he is not presumed to engage for extraordinary skill or for extraordinary dilligence and care; that he is not responsible for errors of judgment or mere mistakes in matters of reasonable doubt and uncertainty.

Of malpractice the author points out that there are three divisions. The first division is classified as willful malpractice, or willful acts of the physician or surgeon toward a person under his care, the result being death or injury to the person. The second, acts forbidden by the statutes on the part of the physician or surgeon toward a person under his care, by which such person may suffer injury or death. The third, negligent acts upon the part of the physician or surgeon in treating a person by which such person may suffer death or unnecessary injury.

It will be of importance to note that the first and second divisions include the class of cases which makes the physician or surgeon liable to punishment in a criminal prosecution. The third division treats of that class of cases where suits are brought against a physician or surgeon for negligence whereby the person suffers death or unnecessary injury in consequence of such negligence.

It is, as the author points out, the hope of gaining money, generally under the prompting of some unscrupulous attorney, that induces patients to bring this class of cases into court, and such cases have multiplied with alarming frequency in late years. The only courses open to the physician when such a suit is instituted are either arbitration or litigation. The former the author opposes in all cases as being held by most people to imply either negligence, incompetence, or cowardice.

On the other hand, in court there is but little guarantee that, no matter how right the physician may feel himself to be, his defense will be successful. The sympathy of the jury is ordinarily with the plaintiff. He then records some cases, including an experience of his own, as tending to show the importance of putting forward all the proof possible of the propriety of his action, and he winds up with the following useful suggestions: 1. When called upon to defend such cases, secure the ablest legal counsel it is possible to obtain, for in this as in all else the cheaper in the beginning may prove the more expensive in the ending. 2. Obtain all the evidence possible to sustain your case. It is evidence, and not sympathy, that will win where suits are to be tried in courts of justice. 3. Do not arbitrate or settle a case for any amount, it matters not how small the amount may be, as when you consent to do this you pay money out of your own pocket to convict yourself. 4. Do not allow a case, if you can help it, to be continued from one court to another until your best and most reliable evidence is lost. 5. Do not reveal to the outside world what you propose to introduce as evidence. 6. Always take the plaintiff's deposition as soon as suit is entered against you. It may serve a double purpose. It will surely give you an insight into the case you are called upon to defend, and, should the plaintiff fail to remember his evidence given in the deposition, it may weaken his testimony given in the court during the trial of the cause. The law gives the defendant the opportunity to take the plaintiff's deposition in all suits for damages.—*New York Medical Journal.*

